# Hand preference in referential gestures: Relationships to accessing words for speaking in monolingual and bilingual children

**DOI:** 10.1002/brb3.2121

**Published:** 2021-06-17

**Authors:** Elena Nicoladis, Bryce S. Dueck, Shiva Zarezadehkheibari

**Affiliations:** ^1^ Department of Psychology University of Alberta Edmonton Canada; ^2^ Department of Educational Psychology University of Alberta Edmonton Canada

**Keywords:** Bilingualism, gesture, hand preference, semantic fluency, visuospatial short‐term memory, vocabulary

## Abstract

**Introduction:**

Infants’ right‐hand preference for pointing is associated with higher vocabulary. It is not clear whether the link between right‐hand preference for gesturing and language persists into the preschool years. The primary purpose of the present study was to test whether preschool children's hand preference for referential gestures was associated with their language abilities. Secondarily, we predicted that the children's right‐hand preference would be negatively associated with their visuospatial abilities. We also predicted that monolingual children would show a strong right‐hand preference while bilinguals might show a reduced right‐hand preference.

**Methods:**

Monolingual and bilingual children between the ages of four and six years did a storytelling task. Their referential gestures were coded for hand use (right, left, both). We measured language skills (receptive vocabulary, semantic fluency).

**Results:**

We found no difference between bilinguals and monolinguals on hand preference. Semantic fluency was a positive predictor and vocabulary a negative predictor of right‐hand preference. Children's visuospatial abilities were not a predictor of right‐hand preference.

**Conclusion:**

These results suggest that right‐hand preference may help children select semantically appropriate words out of their existing vocabulary. In other words, this preference may be related to children's construction of the message that they would like to produce. The association between hand preference and language skills persists into the preschool years.

## Graphical abstract

Preschool children who use their right hand more when gesturing score lower on a standardized vocabulary test but are better at generating examples of a given semantic category (like "animals"). These results could suggest that use of the right hand in gesturing helps children choose particular words for speaking.

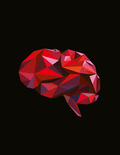

## 1 INTRODUCTION

In this study, gestures refer to communicative hand and arm movements, such as pointing, giving a thumbs‐up to indicate “okay,” or mimicking the hand movement of hammering to communicate hammering (Kita et al., 2017; McNeill, 1992, 2005). Gestures and speech are strongly linked in processing (Kita et al., 2017; McNeill, 1992). For example, Gentilucci and Volta (2008) found that both speech and gestures are controlled by the same left‐hemisphere dominant motor system. Since the left hemisphere controls the right side of the body, it is not surprising that adults tend to produce gestures mostly with their right hand (Corina et al., 1992; Foundas et al., 1995; Thomas Dalby et al., 1980). Similarly, infants tend to prefer their right hand when pointing to draw another person's attention to something (Cochon & Vauclair, 2010; Esseily et al., 2011; Jacquet et al., 2011). Moreover, several studies have shown that infants’ degree of preference for pointing with the right hand is related to vocabulary (Cochet et al., 2011; Esseily et al., 2011; Hamilton et al., 2000; Mumford & Kita, 2016; Nelson et al., 2014). Nelson et al. (2014) found that infants who showed a consistent right‐hand preference in pointing in infancy had higher vocabulary scores as toddlers than infants who showed an inconsistent hand preference in pointing.

As children get older, it is not clear that the link between hand preference in gesturing and language persists (see review in Cochet, 2016). For example, one study with three‐ to five‐year‐olds showed no correlation between productive language abilities and right‐hand preference for either pointing or symbolic gestures (like waving or hushing; Cochet et al., 2015). There are several possible explanations for the lack of evidence to date for a correlation between preschool children's hand preference for gestures and language abilities. One possible explanation is that in the preschool years, children start to produce more representational gestures (Cameron & Xu, 2011), gestures that represent the referent by the movement and/or hand shape (Gliga & Csibra, 2009). Representational gestures have been strongly linked with accessing the appropriate words to convey a message (Feyereisen, 2006). For example, Pine et al. (2007) showed that preschool children had difficulty retrieving words when their hands were immobilized to prevent gestures. It is possible that, in the preschool years, hand preference in gesturing is linked with accessing words for production. Another possible reason that preschool children's hand preference in gesturing has not been linked with their language abilities is that representational gestures, in addition to showing a link with verbal abilities in adults, also reflect visuospatial processing (Kita et al., 2017; Lausberg & Kita, 2003). For example, Chu and Kita (2011) showed that when people had difficulty solving spatial problems, they produced representational gestures to help their reasoning. It is possible that preschool children's hand preference is related to their visuospatial abilities.

The primary purpose of the present study is to test whether we see a relationship between right‐hand preference for gesturing and language abilities in preschool children while telling a story. The gestures produced by the children in this context were referential gestures, communicative hand movements that refer, either deictically, as in pointing, or by representing the referent as in tracing the path of movement of a character (Gliga & Csibra, 2009). We predicted that the greater the right‐hand preference in producing referential gestures, the stronger the children's lexical access abilities. This prediction is premised on the assumption of left hemispheric specialization for processing both gestures and some aspects of language (Gentilucci & Volta, 2008).

In most people, the left hemisphere has been shown to specialize in processing language, such as performing a vocabulary test (Goodglass et al., 1993; Reitan et al., 1988) and generating words from a particular semantic category, as in a verbal semantic fluency task (Gutierrez‐Sigut et al., 2015). In contrast, the right hemisphere has been shown to specialize in the processing of visuospatial information (Davidson & Hugdahl, 1993; for review, see Hugdahl, 2011). Evidence for the lateralization of language and visuospatial functions includes studies of unilateral brain damage (Milner, 1974), neuroanatomy (Galaburda et al., 1978; Geschwind & Levitski, 1968), stimulation of the cortex (Ojemann & Mateer, 1979; Penfield & Roberts, 1959), event‐related potentials (Mills et al., 1993), and split‐brain patients (Gazzaniga et al., 1965; Levy et al., 1972; Zaidel, 1978).

Although these studies lend support for hemispheric specialization, they should not be interpreted as meaning that the left hemisphere is solely responsible for processing language information while the right hemisphere solely for visuospatial information. For example, some studies have shown that the left hemisphere plays a role in various visuospatial tasks (Mehta & Newcombe, 1991). Similarly, the right hemisphere can also perform a variety of language functions, though not to the same degree as the left hemisphere (Zaidel, 1978). Moreover, some language measures show greater right‐hemisphere involvement than others. For example, vocabulary is strongly left‐lateralized while semantic fluency and narrative tasks show greater right‐hemisphere involvement (Baldo et al., 2010; Birn et al., 2010; Brownell et al., 1995; Chiarello, 1985; Goulet et al., 1997; N'Kaoua et al., 2001; Reitan et al., 1988).

In the present study, we included three measures of children's language ability: receptive vocabulary scores, semantic fluency, and lexical diversity in storytelling (or “word types”). While these measures are often highly correlated (Luo et al., 2010; Nicoladis & Jiang, 2018; Sauzeon et al., 2004), the latter two measures reflect children's ability to access words for production. We predicted that all language measures would show positive relationships with right‐hand preference in gesturing, but particularly the lexical access measures.

In addition, to test hand preference for referential gestures and language abilities, a secondary purpose of this study was to test for hand preference and visuospatial abilities. Referential gestures can rely heavily on visuospatial processing (Hostetter et al., 2007; Wagner Cook et al., 2008). One study with adults’ storytelling found no right‐hand preference in referential gestures (Lausberg & Kita, 2003). The authors of that study argued that referential gestures reflect visuospatial processing, since participants tended to gesture with the hand corresponding to the side of the screen on which they had seen the referent. In the present study, we tested whether there was an association between left‐hand preference in referential gesture production and visuospatial skills. If so, there would be a negative relationship between right‐hand preference for gesturing and visuospatial skills.

This study included both monolingual and bilingual children. There is some evidence that bilinguals’ language lateralization might differ from monolinguals’ (Obler et al., 1982; Vaid & Genesee, 1980). Specifically, bilinguals may sometimes show greater right‐hemisphere involvement in language processing when compared to monolinguals (Albert & Obler, 1978; Genesee et al., 1978; Soares & Grosjean, 1981; Wesche & Schneiderman, 1982; Hull & Vaid, 2007; Proverbio et al., 2007). Furthermore, some studies have shown that visuospatial memory is more strongly related to language processing among bilingual children than among monolingual children (Gangopadhyay et al., 2016), perhaps because of the greater right‐hemisphere involvement in language processing for the bilinguals. The age of second language acquisition may affect cerebral lateralization. In meta‐analyses, Hull and Vaid (2007) found that bilinguals who acquired both language by six years of age showed bilateral hemispheric involvement for both languages, whereas bilinguals who acquired their second language after six showed left‐hemisphere dominance for both languages (see also Evans et al., 2002). In the present study, since all the bilingual children started acquiring both languages before the age of three years, the bilinguals may use left‐handed gestures more frequently than the monolinguals. Visuospatial abilities might also be more strongly related to bilinguals’ hand preference than language abilities.

Some previous studies have shown bilingual disadvantages in language abilities, such as vocabulary and semantic fluency (Gollan & Kroll, 2001; Kormi‐Nouri et al., 2012; Oller et al., 2007), and bilingual advantages in visuospatial abilities (Bialystok 2010; Blom et al. 2014; Delcenserie & Genesee, 2017; Morales et al., 2013). However, some studies have shown that bilingual children can produce just as many words on a semantic fluency task (Friesen et al., 2015) and just as many different words in telling a story as monolingual children (Peets & Bialystok, 2015). We test for differences between monolingual and bilingual children on language and visuospatial abilities in the present study.

### 1.1 This study

The primary purpose of this study was to test whether preschool children's right‐hand preference in gesturing was related to their language abilities, particularly lexical access. To address this hypothesis, we included three measures of children's language ability: vocabulary scores, semantic fluency, and the number of different words generated when telling a story (or word types). We expected all language measures to be positively correlated with right‐hand preference in gesturing, particularly the lexical access measures (i.e., semantic fluency and word types).

The secondary purpose of this study was to test whether children's hand preference in gesturing was related to their visuospatial abilities. We predicted that visuospatial abilities might be negatively correlated with right‐hand preference (i.e., positively correlated with left‐hand preference).

Both bilingual and monolingual children participated in this study. Some previous studies have shown that bilinguals might have greater right‐hemisphere involvement in language processing than monolinguals (Obler et al., 1982). Before addressing the main predictions in this study, we first compared the hand preference in bilinguals and monolinguals. Bilingual children might show less of a right‐handed preference in gesturing than monolingual children. If so, then it would be important to analyze the predictors of hand preference separately for bilingual and monolingual children.

Previous studies with preschool children have shown that only about half the children produce referential gestures when telling a story (Laurent et al., 2020). Children who spontaneously gesture when telling a story might differ from children who do not gesture on visuospatial abilities. One study found that gesturers have lower visuospatial abilities than nongesturers (Galati et al., 2018). In contrast, other studies have shown that gesturers tell longer stories than nongesturers (Laurent et al., 2020; Nicoladis et al., 2016). Researchers have generally interpreted these results to suggest that gesturers have stronger visuospatial skills than nongesturers, since visuospatial abilities are required to tell a long story from memory. In the present study, we test whether there are differences between gesturers and nongesturers on language and visuospatial abilities.

## 2 METHOD

### 2.1 Participants

A total of 134 children (66 females) were included in the analyses for this study: 61 English monolinguals, 46 French–English bilinguals, and 27 Mandarin–English bilinguals. Twenty‐three other children (six English monolinguals, four French–English bilinguals, and 13 Mandarin–English bilinguals) participated in this study but did not tell a story so their gestures could not be coded: Their data were not included in any of the analyses. All children were deemed to have no developmental delays by their parents or caregivers. The children were recruited through day cares or by word of mouth in Edmonton, Alberta, Canada. This is an English‐majority‐language part of Canada. Data from these children have been published before (Nicoladis & Jiang, 2018; Nicoladis & Wiebe, 2020), but all the present analyses were new. All children were between the ages of 36 and 85 months with an average age of 61.8 months (*SD* = 8.6). There was a significant difference between language groups on age, *F* (2, 131) = 6.35, *p* = .002, η^2^_p_ = 0.088. LSD post hoc tests showed that the Mandarin–English bilinguals were significantly older (*M* = 66.4 months, *SD* = 9.5) than the English monolinguals (*M* = 59.7, *SD* = 7.8, *p* = .001) and the French–English bilinguals (*M* = 62.0, *SD* = 8.1, *p* = .03). For that reason, we will take age into account in our analyses. A first pass set of analyses revealed no differences by sex on any of our dependent variables (cf. Saucier & Ellis, 2001, who reported lateralization differences between adult men and women). We therefore did not include sex in the analyses we present here.

The English monolingual children had, at most, minimal exposure to any language other than English (e.g., some children could count to ten in French or Spanish). The French–English bilingual children had heard both languages from birth, with at least one parent addressing the child in French and childcare in French. These children can be characterized as simultaneous bilinguals. The Mandarin–English bilingual children, in contrast, are best characterized as early sequential bilinguals. They all heard Mandarin Chinese from both parents from birth. Their age of onset of English acquisition varied between the age of one and three years of age, usually coinciding with their start of day care or preschool in English.

Approximately half of the children (*N* = 70) produced at least one referential gesture while telling a story (29 English monolinguals, 26 French–English bilinguals, and 15 Mandarin–English bilinguals). The other children (*N* = 64) told a story but produced no referential gestures (32 English monolinguals, 20 French–English bilinguals, and 12 Mandarin–English bilinguals). We test whether there are any differences between the gesturers and nongesturers on age, visuospatial short‐term memory, and language measures.

### 2.2 Materials

#### 2.2.1 Storytelling

To elicit gestures, we used two Pink Panther cartoons (total running time of 8 min 33 s) used in previous research to elicit gestures (e.g., Nicoladis et al., 2009). Each child was asked by the experimenter to recall what he/she saw during the two cartoons. The experimenter claimed not to have seen the cartoon before. The bilinguals did this task in both their languages, on different days usually separated by a week, with the order of the language sessions counterbalanced. The experimenter in each language session was a native speaker of the target language of the session and was a different person for each language session. The bilinguals watched the same cartoon in both language sessions. In the present study, we analyze only the English stories, for consistency across the groups. The storytelling task was recorded using a video camera and moved to a computer where it was transcribed for speech and coded for referential gestures (described further below).

For this task, we also counted the number of unique words (i.e., the word types) that the children used in order to tell the story (see Table 1 for summary). The difference between language groups on word types did not reach significance, *F* (2, 131) = 2.44, *p* = .09, η^2^_p_ = 0.037. For the bilinguals, there was no difference on word types by language session (Nicoladis & Jiang, 2018; Nicoladis & Wiebe, 2020).

**TABLE 1:** Averages (SDs) of all measures in this study

	English monolinguals	French–English bilinguals	Mandarin–English bilinguals	All children
Age in months	59.7 (7.8)	62.0 (8.1)	66.4 (9.5)	61.8 (8.6)
VS‐STM	3.3 (1.2)	3.5 (0.9)	2.9 (1.7)	3.3 (1.2)
PPVT (raw scores)	84.8 (27.0)	69.5 (19.9)	34.3 (22.6)	69.2 (30.3)
SF	14.1 (6.5)	19.0 (9.9)	13.8 (6.5)	15.7 (8.0)
Word types	39.0 (20.0)	45.4 (21.0)	53.0 (38.2)	44.0 (25.4)
RH preference	44.2% (43.0%)	57.5% (41.7%)	40.5% (46.2%)	48.4% (43.2%)
HI	0.188 (0.932)	0.437 (0.833)	−0.029 (0.940)	0.242 (0.900)

#### 2.2.2 Visuospatial short‐term memory

To assess visuospatial short‐term memory (VS‐STM), we used the Corsi block test (Lezak, 1983) to measure children's short‐term visuospatial memory. For the Corsi block task, each child was shown a display of seven blocks on a computer screen. The experimenter touched a series of blocks, starting with two blocks, and asked the child to repeat the sequence. If the child repeated the sequence correctly, the experimenter added one more block onto the next trial. The measure that was used was the highest number of blocks touched in the correct order. See Table 1 for the average scores on the VS‐STM task. The difference between the three language groups on visuospatial short‐term memory did not reach significance, *F* (2, 129) = 2.55, *p* = .08, η^2^_p_ = 0.038.

#### 2.2.3 English vocabulary

We used the Peabody Picture Vocabulary Test Version IIIA (PPVT) to measure children's receptive vocabulary in English (Dunn & Dunn, 1997). The test was administered according to the tester's manual. During the PPVT, each child was presented with a word and a series of 4 black and white pictures. Each child was asked to point to the picture or to indicate the number of the picture (1–4) that corresponded with the examiner's word. Because we were interested in the size of children's vocabulary in English, we included the raw scores as our dependent variable in this study (see Table 1). As expected, there was a significant difference between the three groups on raw PPVT scores, *F*(2, 126) = 53.05, *p* < .001, η^2^_p_ = 0.411. Post hoc LSD tests revealed that English monolinguals scored higher on English vocabulary than both bilingual groups (*p*s < 0.001). The French–English bilinguals also scored higher than the Mandarin–English bilingual group (*p* < .001).

#### 2.2.4 Verbal semantic fluency

For the verbal semantic fluency task, each child was presented with three categories: Clothes, Animals, and Food/Drink. Each child was given a minute to generate as many examples as possible for each category before moving onto the next category. Verbal semantic fluency tasks can be used to measure lexical knowledge and lexical retrieval (Weckerly et al., 2001). The total number of correct words generated has been shown to be related to language ability, particularly word knowledge (i.e., vocabulary size; Ruff et al., 1997; Sergeant et al., 2002). In this study, we included the total number of unique words that were valid exemplars of the category (see Table 1).

There was a significant difference between the three groups on semantic fluency, *F*(2, 126) = 5.76, *p* = .004, η^2^_p_ = 0.085. LSD post hoc tests revealed, surprisingly, that the French–English bilinguals had higher semantic fluency than the English monolinguals (*p* = .008) and Mandarin–English bilinguals (*p* = .001) and no other differences between groups.

### 2.3 Coding

#### 2.3.1 Gesture coding

Children's referential gestures were coded. Referential gestures were defined as communicative hand movements that conveyed semantic meaning. We included as referential gestures those that would be considered iconic, deictic, and conventional according to McNeill’s (1992) classification system. Iconic gestures resemble the referent in some way, such as the child miming the Pinker Panther pushing buttons. Deictic gestures referred to a static location, such as the child pointing to the location of the Pink Panther's house already established in discourse. Conventional gestures referred to gestures that are commonly recognized within a linguistic community such as a child holding up their palms to indicate, “I don't know.”

#### 2.3.2 Hand preference coding

Children's referential gestures were coded for hand use: right, left, or both hands (see the Appendix for summary of numbers by language group). For each child, we calculated the percentage of gestures (out of the total number of gestures) that were produced with the right hand. We refer to this measure as the right‐hand preference (see Table 1 for descriptive data). Many previous studies on hand preference (Cochet et al., 2011; Cochet & Vauclair, 2010, 2014) use the handedness index (HI), calculated as the number of right‐hand gestures minus the number of left‐hand gestures, divided by the total number of unimanual gestures. In the present study, we also present the descriptive statistics for the HI, to allow easier comparison with previous studies (see Table 1). It is important to keep in mind that the right‐hand preference and the HI were highly correlated among the children in this study, *r* (68) = 0.851, *p* < .001.

### 2.4 Analyses

All statistical analyses were carried out in SPSS.

## 3 RESULTS

### 3.1 Differences between language groups on right‐hand preference

Before proceeding to the main analyses, we compared the three language groups on right‐hand preference in gesturing. To test whether there were differences between the language groups on right‐hand preference, we focused on the 70 children who produced at least one referential gesture. The English monolinguals produced an average of 4.6 (*SD* = 5.5) referential gestures, the French–English bilinguals 2.6 (*SD* = 2.7), and the Mandarin–English bilinguals 3.9 (*SD* = 4.6). There was no significant difference between the three groups on the number of gestures produced, *F*(2, 68) = 0.57, *p* = .57, η^2^_p_ = 0.008.

We predicted that the bilingual children would show less of a right‐hand preference in gesturing than monolingual children. To test that prediction, we first compared the language groups on right‐hand preference (see Table 1). There were no differences between language groups on the percentage of right‐handed gestures, *F*(2, 66) = 0.90, *p* = .41, η^2^_p_ = 0.026.

In sum, there are no differences on the number of gestures or on right‐handed preference by language group. We therefore combined the language groups for the remaining analyses.

### 3.2 Predictors of right‐hand preference

Table 2 summarizes the correlations between age, the language measures, and degree of right‐hand preference for the 70 children who produced at least one referential gesture (below the diagonal). For the gesturers, the only variable to correlate significantly with right‐hand preference was semantic fluency, although word types also showed a positive correlation. Surprisingly, the PPVT scores were negatively (although not significantly) correlated with the gesturers’ right‐hand preference. For the nongesturers (in gray, above the diagonal), the language measures and VS‐STM showed many strong positive intercorrelations.

**TABLE 2:** Correlations between age, visuospatial short‐term memory, language measures, and right‐hand preference

	Age	VS‐STM	PPVT	SF	Word types
Age	—	0.374**	−0.024	0.450**	0.333**
VS‐STM	0.146	—	0.321**	0.249*	0.440**
PPVT	0.142	0.416**	—	0.342**	0.134
Semantic fluency	0.125	0.083	0.182	—	0.225
Word types	0.439**	0.071	0.022	0.181	—
RH preference	0.037	0.114	−0.146	0.244*	0.165
HI	−0.057	0.036	−0.158	0.248*	0.041

Note

Correlations for nongesturers above the diagonal (in gray) and gesturers below.

Abbreviations: HI, handedness index; PPVT, Peabody Picture Vocabulary Test scores; RH preference, right‐hand preference in gesturing; VS‐STM, visuospatial short‐term memory.

* *p* < .05.

** *p* < .01.

Given the colinearity of some of the language measures (see Table 2), we next predicted the percentage of right‐handed gestures with VS‐STM, vocabulary (PPVT), semantic fluency, and word types with a linear regression analysis. We controlled for age by entering age in months as the first step of the regression. The first step did not reach significance, R^2^ = 0.003, *F* (1, 64) = 0.17, *p* = .68, beta = 0.052. The second step, with the remaining predictors, approached significance, R^2^ = 0.154, *F* (5, 60) = 2.18, *p* = .068. The results of the second step of the analysis are presented in Table 3. There were two significant predictors of right‐hand preference: vocabulary (a negative predictor) and semantic fluency (a positive predictor).

**TABLE 3:** Predictors of right‐hand preference

Predictors	Coefficients		
	ΔR	ß	*p*
	0.151	—	.068
VS‐STM	—	0.184	.171
PPVT	—	−0.277	.043*
Semantic fluency	—	0.275	.028*
Word types	—	0.153	.261

Abbreviations: PPVT = Peabody Picture Vocabulary Test scores; VS‐STM = visuospatial short‐term memory.

### 3.3 Differences between gesturers and nongesturers

Table 4 summarizes the average (*SD*) age, VS‐STM, and language measures for the gesturers and the nongesturers. Independent‐samples *t* tests revealed only a significant difference on the number of word types: The gesturers produced more word types when telling a story than the nongesturers.

**TABLE 4:** Average (*SD*) of visuospatial short‐term memory, English vocabulary, semantic fluency, and word types for gesturers and nongesturers

	Gesturers (*N* = 70)	Nongesturers (*N* = 64)	*p*‐value for *t* test
Age in months	61.9 (8.1)	61.7 (9.2)	.90
VS‐STM	3.4 (1.2)	3.2 (1.2)	.26
PPVT	72.0 (30.1)	66.2 (30.5)	.27
Semantic fluency	15.7 (8.3)	15.7 (7.8)	.99
Word types	53.9 (27.2)	33.2 (18.0)	<.001

Abbreviations: PPVT, Peabody Picture Vocabulary Test scores; VS‐STM, visuospatial short‐term memory.

## 4 DISCUSSION

The primary purpose of this study was to test the hypothesis that right‐hand preference would correlate with language abilities (as Nelson et al., 2014, found for infants and pointing). We found some support for that prediction: Verbal semantic fluency was a positive predictor of right‐hand preference. Surprisingly, vocabulary scores were a significant negative predictor of right‐hand preference. This result is surprising because previous studies have often shown vocabulary is highly positively correlated with semantic fluency and word types in storytelling (Luo et al., 2010; Nicoladis & Jiang, 2018; Sauzeon et al., 2004) and because vocabulary generally shows greater left‐hemisphere involvement than other language measures (Birn et al., 2010). In the present study, the three language measures were not strongly correlated among the gesturers (see Table 2).

While surprising, this pattern of results may reveal something novel about the connection between right‐hand preference for gesturing and language. Right‐hand preference may be related to the active construction of the message to be produced out of the (receptive) vocabulary available. Children whose vocabulary in the target language is low and who use their right hands to gesture while searching for how to express their message may end up accessing many different words in the target semantic area. In support of this explanation, gestures are often produced when speakers are considering options for conceptualization (Kita & Davies, 2009). One way to test this explanation is to immobilize only children's right hand or only children's left hand during a semantic fluency task. We predict that immobilizing children's right hand will interfere more with semantic fluency than immobilizing their left hand, particularly when their (receptive) vocabularies are low. Pine et al., (2007) that immobilizing both of children's hands interfered with lexical access. Similarly, Laurent et al., (2020) found that children whose hands were restricted (both of them) told shorter stories than children who could move their hands. Neither study tested whether the immobilization of one hand contributed more to the interference than the immobilization of the other.

If our interpretation is correct, then these results may have come to light in part because of the inclusion of both bilingual and monolingual children. Previous research has shown that bilinguals often score lower on vocabulary tests than monolingual children (Bialystok et al., 2010; this study), but can sometimes produce just as many words on a semantic fluency task (Friesen et al., 2015; this study) and in telling a story (Peets & Bialystok, 2015; this study). Thus, some bilingual children can generate more semantically appropriate words than might be predicted by their vocabulary scores in that language. The present results suggest that this same ability could be present in some monolingual children as well, since many of the children included in the regression analysis were monolingual. Future studies can test whether children's hand preference in gesturing contributes to this constructive ability.

A secondary purpose of this study was to test whether visuospatial abilities were related to hand preference in gesturing. In addition to language abilities, we had also predicted that visuospatial abilities would be linked to children's hand preference for gesturing. Previous research has shown right‐hemisphere specialization for visuospatial tasks (Hugdahl, 2011). We therefore predicted that children's degree of left‐hand preference would be related to their visuospatial abilities. In this study, we found no relationship between visuospatial abilities and hand preference. Among adults, Lausberg and Kita (2003) found that hand preference in referential gestures while doing a cartoon retell task was related to the side of the screen that they had seen actions in the cartoon unfold. Future studies can explore under what circumstances children produce left‐handed referential gestures.

In sum, these results suggest that children's right‐hand preference in producing referential gesturing is related to constructing appropriate ways of phrasing their message when their vocabulary is small. We found no evidence linking their hand preference in gesturing with visuospatial abilities.

### 4.1 Bilingualism

This study included both bilingual and monolingual children. Some previous research has shown that bilinguals have higher right‐hemisphere involvement in language processing when compared to monolinguals (e.g., Proverbio et al., 2007). We therefore predicted that bilingual children would show a reduced right‐hand preference when producing referential gestures than monolinguals. Contrary to our prediction, our results showed no difference between monolinguals and bilinguals on the percentage of right‐handed gestures. One possible reason for this apparent contradiction is that the previous research on the hemispheric specialization of bilinguals’ language processing has mostly focused on receptive language, rather than language production as we did in the present study. Future studies can test that interpretation.

### 4.2 Gesturers versus nongesturers

About half the children in this study did not gesture when telling a story. Researchers have argued that gesturers may differ from nongesturers on visuospatial abilities (Galati et al., 2018; Laurent et al., 2020). In this study, we did not find any differences between gesturers and nongesturers on visuospatial ability (see Table 4). The gesturers did use more word types to tell the story than the nongesturers did. Curiously, the nongesturers showed higher intercorrelations between the different language measures and visuospatial abilities (Table 2, shaded part) than the gesturers (Table 2, unshaded part). It is not entirely clear to us why these intercorrelations should differ between the two groups. Future studies can test whether these results replicate and, if so, why there might be different patterns of intercorrelations between children who spontaneously gesture and those who do not.

### 4.3 Degree of hand preference

In this study, the children did not show an overwhelming right‐hand preference for referential gestures: They averaged only 48% right‐handed gestures (out of gestures produced with left, right, or both hands). In contrast, adults showed a very high right‐handed preference for pointing and for referential gestures when asked to say what they would do in an imaginary scenario (Cochet & Vauclair, 2014). However, the present results are remarkably similar to the adults in Lausberg and Kita (2003). In that study, the adults did not show a right‐hand preference for referential gestures when telling a story. Taken together, these results suggest that the degree of right‐hand preference in gesturing may be somewhat dependent on the task. Future studies could consider the degree to which different tasks require actively constructing a message based on available vocabulary.

### 4.4 Limitations and future directions

While we have already mentioned a number of limitations and suggestions for future research, we would like to point out another limitation that we have not yet discussed. In the present study, we only included in the analyses the bilingual children's language abilities in English and not their other language. We opted to take this approach so that we could compare the same measures for the English monolinguals and for the bilingual children. Future studies can test whether bilinguals’ language ability in both of their languages is related to their hand preference in gesturing.

## 5 CONCLUSION

This study has shown that the relationship between hand preference in gestures and language abilities extends beyond infancy (Esseily et al., 2011; Nelson et al., 2014). We found that preschool children's right‐hand preference in producing referential gesture was negatively related to vocabulary scores and positively related to their semantic fluency. We interpreted these results to mean that producing right‐hand referential gestures aids the constructive search for semantically appropriate words out of the existing vocabulary. Future studies are needed to test that interpretation. Of particular, importance will be longitudinal studies, in order to test whether right‐hand preference leads to enhanced language skills (as shown in Nelson et al., 2014) or vice versa.

Author contribution: EN posed the research questions, collected the data, did the final analyses, and wrote the first draft of the manuscript. BSD organized the data, did an initial review of the literature, and edited the manuscript. SZ did the preliminary analyses and edited the manuscript. All authors have approved this version of the manuscript.

## CONFLICT OF INTEREST

The authors declare no conflict of interest.

### PEER REVIEW

The peer review history for this article is available at https://publons.com/publon/10.1002/brb3.2121.

## Data Availability

The data that support the findings of this study are available from the corresponding author upon reasonable request.

## APPENDIX 1 Averages (SDs) number of referential gestures

**Table:**

	English monolinguals	French–English bilinguals	Mandarin–English bilinguals	All children
# Right hand	1.8 (2.5)	1.3 (1.4)	2.5 (4.8)	1.8 (2.8)
# Left hand	0.9 (2.2)	0.6 (1.1)	1.0 (1.6)	0.8 (1.7)
# Both hands	1.9 (3.2)	0.7 (1.4)	0.4 (0.8)	1.1 (2.3)
